# Factors associated with the uptake of immediate postpartum intrauterine contraceptive devices (PPIUCD) in Rwanda: a mixed methods study

**DOI:** 10.1186/s12884-020-03337-5

**Published:** 2020-10-27

**Authors:** Chris Adrien Kanakuze, Dan Kabonge Kaye, Priscilla Musabirema, Pascal Nkubito, Scovia Nalugo Mbalinda

**Affiliations:** 1 Faculty of Health Sciences, Kibogora Polytechnic, P.O. Box: 31, Rusizi, Rwanda; 2 Department of Nursing, Makerere University, College of Health Sciences, School of Health Sciences, P.O. Box 7072, Kampala, Uganda; 3 Department of Obstetrics and Gynecology, Makerere University, College of Health Sciences, School of Medicine, Kampala, Uganda; 4 Midwifery department, Faculty of Nursing Science, University of Rwanda, Kigali, Rwanda; 5 Department of Obstetrics and Gynecology, Muhima Hospital, Kigali, Rwanda

**Keywords:** Immediate postpartum women, PPIUCD, Long-acting family planning method

## Abstract

**Background:**

Rwanda has a high unmet need for family planning which could be reduced by improving access to postpartum intrauterine contraceptives device (PPIUCD) insertion. The objective of the study was to assess the prevalence and factors associated with the uptake of PPIUCD among postpartum women in Muhima Hospital.

**Methods:**

A concurrent mixed-method study was used. Three hundred eight three (383) immediate postpartum mothers, and 10 health services providers were interviewed using a structured questionnaire and in-depth interviews respectively. Logistics regression was done to assess for factors associated with PPIUCD uptake and thematic analysis was used for qualitative data.

**Results:**

The prevalence for PPIUCD use was 28.1%, women who had spontaneous vaginal delivery were more likely to take up PPIUCD (Adjusted Odds Ratio (AOR) 2.623, 95% CI = 2.017–6.507 compared to those who had cesarean section; women who received PPIUCD counselling during the antenatal period were more likely to use PPIUCD ((AOR 2.072, 95% CI = 1.018–4.218) as compared to those who didn’t receive any form of counselling; mothers who received spouse approval were more likely to use PPIUCD (AOR 2.591,95% CI = 1.485–4.492); as compared to those who didn’t receive any spousal approval; women who had more than one child were more likely to use PPIUCD (AOR =2.265, 95% CI = 1.472–3.163) as compared to prime gravida; Mothers with birth to pregnancy interval less than two years were more likely to use PPIUCD (AOR =2.123, CI =1.477–2.706) as compared to those who had birth to pregnancy interval more than 2 years. From the qualitative findings, health education of mothers and partners on PPIUCD, training of health care providers, and availability of supplies to provide PPIUCD influenced the use of PPIUCD.

**Conclusion:**

The acceptability to use for PPIUCD was high in this population. PPIUCD uptake was associated with normal birth, PPIUCD counselling, spousal approval, parity, birth interval, level of education. Health education of mothers and partners on PPIUCD, training of health providers, and availability of supplies to provide PPIUCD influenced uptake of PPIUCD.

## Background

Postpartum family planning (PPFP) is the prevention of unintended pregnancy and closed pregnancy through the first 12 months following childbirth and is recognized as a key life-saving intervention for mothers and their children [1]. Globally, it is estimated that 214 million women of reproductive age in low-income countries want to avoid pregnancy but are not using a modern contraceptive [2]. Data from 27 countries, shows 95% of women who are 0–12 months postpartum want to avoid pregnancy in the next 24 months; but 70% of them are not using contraception [1, 3]. In sub-Saharan countries, postpartum contraceptive intrauterine device (PPIUCD) still represents a small proportion of contraceptive service delivery [4, 5]. The World Health Organization (WHO) recommended that birth to pregnancy interval should be 24 months [4]; since short birth intervals are associated with adverse pregnancy outcomes such as induced abortions, miscarriage, preterm births, neonatal and child mortalities, stillbirths, and maternal depletion syndrome [5]. Research has demonstrated that long-acting methods such as intrauterine devices (IUDs) are a cost-effective and sustainable way of reducing the unmet need and unintended pregnancy in low resources settings [6].

PPIUCD is one of the contraceptive methods which is safe and highly effective reliable and inexpensive, non-hormonal, reversible, and long-acting contraceptive [7]. The WHO recommended can be initiated during the immediate postpartum period and it cannot interfere with breastfeeding [6, 8]. The PPIUCD has shown that when it is initiated after delivery it can improve maternal and newborn health by preventing obstetrics complications such as maternal and newborn mortality and other health-related complications associated with closed spaced pregnancy [9]. The immediate postpartum period is an ideal time for PPIUCD when women are highly motivated to accept family planning methods [10, 11]. The immediate postpartum period is a great opportunity for PPIUCD services providers to introduce the method especially in a setting where women meet with health care providers is difficult due to geographical barriers [10]. An intervention study done in Tanzania aimed to improve women’s access to PPIUCD focused on increasing counselling has revealed that universal counselling and provision of IEC Materials after admission for delivery were associated with high acceptability for PPIUCD [11], this evidence that improving quality of counselling, will result to increase the women’s accessibility to PPIUCD. Frequent exposures to formal health counselling classes and prior discussion with their husband and entire family members could improve the knowledge and the likelihood of acceptance of PPIUCD [12].

Lack of provision for PPIUCD can contribute to the occurrence of unintended pregnancies because most of the women do not return for postnatal services [9]. The early initiation and provision of the postpartum family planning method (PPIUCD) during the immediate postpartum period protect women from unintended pregnancy as the majority of women either resume early sexual activity or have early return to fertility [1, 9].

A systematic review looking the safety and expulsion rates of postpartum IUDs has supported that the insertion of an intrauterine contraceptive within the first 48 h of vaginal or cesarean delivery is safe [13], both vaginal and cesarean section should be encouraged, however early follow up should be encouraged to detect expulsions and tackle commons problems [13].

Another Cochrane systematic review looking at the effectiveness for IUD inserted immediately after placenta delivery showed that the benefits of effective contraceptives immediately after delivery outweigh the disadvantages for increased risks for expulsion [14], there is a need to strengthen the frequent prenatal visits during the third trimester hence it provides the opportunity to discuss the effective contraceptive methods and desired to time for initiation [14]. Clinical follow-up can help detect early expulsion, as can educating women about expulsion signs and symptoms [14].

Rwanda Demographics Health survey showed the women who attend ANC stands at 98% and delivery at facility 91% and family planning in Rwanda is free of charges [13, 14] despite this, there is a high unmet of postpartum family planning at 19% of women with short birth to pregnancy interval, consequently, women don’t achieve their fertility preference which further indicates the need for strengthening PPFP/PPIUD at health facility level [13, 15]. There is a need to reach all postpartum women with an unmet need for family planning.

The Ministry of Health in Rwanda has adopted the recommendations of WHO which stipulate the integration of PPIUCD services into the immediate postpartum care services which indicate attempts to initiate women on postpartum IUD after delivery. This would reduce the unmet need for postpartum family planning and reduce unplanned pregnancies through increasing access to long term FP/IUD for women after childbirth. In Rwanda, however, no study has been done to determine the current utilization and factors associated with the use of PPIUCD.

This study aimed to determine the prevalence and factors associated with use for PPIUCD among postpartum mothers within 48 h after delivery.

## Methods

### Study area and period

The study was conducted at Muhima hospital, Department of Obstetrics and gynecology which is located in Nyarugenge District sector, Kigali city. Muhima is a 128-bed hospital specializing in gynecology and neonatology. This hospital oversees eight functional health centers. Muhima is the training site for all medical schools and schools of nursing and midwifery in Rwanda.

Muhima District Hospital was chosen because it has a high number of deliveries and it is also one of the hospitals where the implementation of PPIUD was initiated. It has an average of more than 500 deliveries monthly.

### The study design

Mixed-method concurrent design was employed to determine the use of PPIUCD and its associated factors. The cross-sectional design involved the use of an interviewer-administered questionnaire which was conducted among women who were in the immediate postpartum period. Immediate postpartum was defined as women who have given have given birth10 minutes after birth up to 48 h of delivery [6] Also, in depth-interviews was conducted among health care providers at Muhima hospital to share their experiences about PPIUD service delivery.

### Study participants and sampling procedures

The study population consisted of women who were in the immediate postpartum care within 48 h after delivery before discharge at Muhima district hospital and Midwives who were working in the labor suite, immediate postpartum ward, and family planning department who were actively involved in the provision of PPIUCD.

The study used a consecutive sampling method to select the eligible participants who were within 48 h after delivery. The participants were approached in the labor ward or postpartum ward where they were invited to take part in the study. The files of women who had delivered were checked to ensure that they had fulfilled the inclusion criteria of the study. The inclusion criteria for PPIUCD were Women aged 15–49 years, delivering either vaginally or by cesarean section, at Muhima hospital from January – February 2019 had received counselling for postpartum contraception and consented for postpartum use who were in the immediate postpartum period after delivery within 48 h of birth and midwives who were working in FP services, labor suite, immediate postpartum ward who were providing FP services and other maternal health services on the days of the study were included in the study.

The exclusion criteria include: The study excluded women who were within10 minutes after childbirth to 48 h after delivery but clinically unstable such as the women who had PPH, Women who have been diagnosed with chorioamnionitis, Women who have a fever ≥37.5 degree Celsius during labor, and delivery, Women who had active genital tract infections or were are at high risk for STD, Women are known to have ruptured membranes for greater than 24 h before delivery. Women who have had 3rd and 4th-degree tears, Women who had been diagnosed to have uterine abnormalities, Health care workers who were not providing the methods.

The participants were informed about the study including providing adequate information regarding the purpose, procedure, benefits, and risks of the study. The potential participants consented before they were interviewed for the study. Women who accepted to be inserted an IUD were considered to have used PPIUCD, while women who declined to use IUD were considered to have not used the PPIUCD.

### Sample size determination

The sample size was powered to determine the prevalence of PPIUD. The sample size was calculated using a prevalence of 48% for contraceptive prevalence, 95% confidence interval, and an error margin of 5%. A total of 383 mothers were recruited for this study [16].

The sample size for factors associated was calculated using α = Type 1 error 5%., Z = the standard normal statistic corresponding to 1.96; β = Type II error as 20%; Odds = 3.1. The odds were derived from literature; Percent of exposed with outcome = 14; Risk/Prevalence ratio = 2.8; Risk/Prevalence difference = 9; Assuming a power of 80%, type 1 error of 5%, type II error of 20%, and odds of 3.10, the sample size of the study was estimated at 374 women. The study adopted a sample size of 383 to increase the statistical power of the study. Therefore, a sample size of 383 women was used in this study. The Fleiss formula in OpenEpi was used for determining the factors associated with the use of PPIUCD. (Ref: Fleiss, Statistical Methods for Rates and Proportions, formulas pages 3.18 & 3.19). The OpenEpi Calculator was used to calculate the sample size and it was accessed from https://www.openepi.com/SampleSize/SSCohort.htm.

### Variables measurements

Data were collected through face-to-face interviewer-administered questionnaires; mixed-method were applied to collect data. The data collected include Social demographic factors; (age, level of education, marital status religious beliefs) knowledge about PPIUCD; Social-cultural factors:, (myths cultural norms, partner, support, peer influence) social-economic factors; (poverty, source of income, occupation), Reproductive factors: (parity, number of living children desired, mode of delivery, fertility desire, side effects of methods), Service delivery related factors; (availability for suppliers and IUCD, health care worker knowledge and skills, access to the health facility, knowledge for health care providers, quality of care delivered to the women, Family planning information and counselling during antenatal care). The prevalence of participants who used PPIUCD was measured. The tools were piloted and pretested before starting data collection to assess appropriateness, content clarity, and comprehensiveness of the questions and time taken to fill the questionnaires.

### Data collection

#### Quantitative data collection

The interviewer-administered structured questionnaires were used to collect quantitative data. The questionnaires included questions regarding the social demographics, knowledge about PPIUD use, social-cultural, social-economic, and reproductive factors.

The study used a consecutive sampling method to select the eligible participants who were within 10 min to 48 h after childbirth. The participants were approached in the labor ward or postpartum ward where they were invited to take part in the study at Muhima hospital. The files of women who had delivered were checked to ensure that they had fulfilled the inclusion criteria of the study. The participants were informed about the study including providing adequate information regarding the purpose, procedure, benefits, and risks of the study. The potential participants consented before they were interviewed for the study. Women who accepted to use an IUD were considered to have used PPIUCD, while women who declined to use IUD were considered to have not used the PPIUCD.

#### Qualitative data collection

In-depth interviews (IDI) were used on one to one to explore the health care provider’s experience regarding PIUCD service provision. An IDI was used because it was able to capture detailed information and offers participants opportunities to share their personal experiences regarding provision for PPIUCD including the mode of supply, the training opportunity, and the institution support through PPIUCD provision. The approach has helped to distinguish an individual’s opinions about provision for PPIUD.

Participants were purposively selected among the team of midwives working in antepartum, labor suite, postpartum, and family planning for the time of data collection. The study purposively selected midwives for an interview until saturation was met, which was reached after interviewing 10 midwives. The IDI was conducted at the workplace in the Muhima hospital.

The IDI was conducted among selected health care providers in a convenient place where she or he was working at the time for data collection. An interview guide containing questions related to experience and practice and their perception regarding provision for PPIUCD was used in this facility-based. The interview was audiotaped and notes were taken by the research assistant.

### Quantitative data analysis

The outcome variables in this analysis are a binary variable for postpartum intrauterine contraceptive use and proportions were used to summarize participants who used PPIUCD. PPIUCD use is defined as any participant who chose IUD as postpartum family planning. Those women who did not choose to use IUD as postpartum contraceptive were classified as non-user. The prevalence of PPIUCD use was determined by dividing the number of women who had accepted to use PPIUCD by the total number of all postpartum women who participated in the study.

To determine the factors associated with the use of PPIUCD, chi-square tests, and binary logistics regression were used. The bivariate analyses were conducted to determine the independent variables that were significantly associated with PPIUCD use. The significant variables were of value less than *p* < .05 at the 95% confidence interval. The variables that were *p* < 0.2 were subjected to multivariate analyses in the binary logistic model to obtain the adjusted odds ratios of the statistically significant variables.

### Qualitative data analysis

The interviews were recorded and transcribed verbatim in Kinyarwanda, after validating the transcription, the typed narratives were then translated into English and verified the accuracy. Analysis of the data was conducted by the primary author and included several iterative steps. Using thematic analysis, the transcripts were reviewed several times, and a set of codes were developed to describe groups of words or categories with similar meanings. The transcripts were then coded and managed using Atlas’s version 7. The grouped categories were refined and used to generate themes emerging from the data. Direct quotations from midwives are presented in italics to highlight key findings.

### Ethical review and approval

Ethical review and approval were obtained from the Higher Degrees and Research Ethics Committee of the College of Health Sciences at Makerere University **#SHSREC REF NO: 2018–045** and Research Ethics Committee of the College of Medicine and Health Sciences University of Rwanda **No 404/CMHS IRB/2018**. The administrative clearance and permissions were obtained from Muhima hospital ethical committee. Written informed consent was obtained from the mothers and the midwives. Participation was voluntary and all the interviews were conducted in private settings to ensure participant’s confidentiality.

## Results

### Social demographic characteristics

Table 1 shows that the mean age of study participants was 28.6 years (SD ± 4.3 years). More than one-half (58.7%) of the respondents had a tertiary level of education and 10.4% had no formal education (Illiterate). Nearly one-half (47.5%) of the participants were Catholic, 82% were legally married, and more than one-third (37%) were employed (37%). More than three-quarters (77.5%) of the women were living in urban areas.

**Table 1:** Social demographic characteristics

Variable	Frequencies (%)
**Age in years (Mean age X = 28.9, SD ± 4.3**	
18–24	94 (24.5%)
25–34	194 (50.7%)
35–40	95 (24.8%)
**Occupation**	
Unemployed	125 (32.6%)
Employed	143 (37.3%)
Student	115 (30%)
**Education level**	
No formal education	42 (10.9%)
Primary	116 (30.28%)
Tertiary	225 (58.7%)
**Religious beliefs**	
Catholic	182 (47.5%)
Protestant	68 (34.7%)
Muslim	133 (17.8%)
**Marital status**	
Single	76 (19.8
Married	307 (80.1%)
**Place of residence**	
Urban	297 (77.5%)
Rural	86 (22.5%)

### Knowledge and use of PPIUCD

Table 2 shows that the majority of the women 324 (84.6%) reported that they had ever heard about the PPIUCD. The reasons for using PPIUCD included the associated fewer side effects (14.9%), non-interference with breastfeeding (10.2%), and is a long-acting method (9.1%). Some of the women did not use the method due to fear of the side effects (10%), inadequate knowledge on the method (12%), and partner’s disapproval (10%) of the method. A significant number of women were informed on PPIUCD used during antenatal care (39.9%), while others got the information during family planning services (37.1%). The majority of women 239 (62.4%) were para 2–5; and those who had normal delivery 62.9% and cesarean section, the proportion using postpartum IUCD among women who were attending Muhima Hospital were determined to be 28.1%.

**Table 2:** Reproductive actors and source of knowledge

Variable	Frequency (%)
**Parity**	
1	91 (23.8%)
2–4	239 (62.4%)
> 5	53 (13.8%)
**Mode of delivery**	
Normal delivery	241 (62.9%)
Cesarean section	142 (37.1%)
**Ever heard PPIUCD**	
Yes	324 (84.6%)
No	59 (15.4%)
**Reason to use PPIUCD**	
Long-acting	35 (9.1%)
Child spacing	15 (3.9%)
Does not interfere with breastfeeding	38 (9.9%)
No hormonal related sides effects	56 (14.9%)
Fewer routine visits	14 (3.9%)
Preference for other methods	225 (58.4%)
**Reason for don’t to use PPIUCD**	
Don’t want contraceptives	30 (8.2%)
Satisfied with the previous method	24 (6.3%)
Afraid of sides effects	76 (20%)
Husband disapproval	31 (10%)
No knowledge about PPIUCD	38 (12%)
Preference for other methods	184 (48.0%)
**Sources of knowledge**	
Media	19 (4.9%)
Antenatal clinic	153 (39.9%)
Family planning clinic	142 (37.1%)
Friends	69 (17.8%)
**Preferred other methods**	
Contraceptives pills	14 (3.6%)
Male condom	19 (5.0%)
Implant	75 (19.5%)
Injectable	31 (8.0%)

### Factors associated with postpartum IUD use

The Table 3 shows that the women who had normal delivery were three times more likely use of PPIUCD (AOR = 3.623; 95% CI 2.017–6.507) compared to cesarean delivery; participants who have PPIUCD counselling were two times more likely to use PPIUCD (AOR =2.072; 95% CI 1.018–4.218) compared their counterpart who did not receive counselling, women who got the Spouse approval were two times more likely using PPIUCD (aOR = 2.583; 95% CI 1.485–4.492) compared with women who did not get approval; participants who have P2-P4 were two times more likely to the use PPIUCD (AOR =2.265; 95% CI 1.472–3.163); pregnancy interval was significantly associated with the use of PPIUCD (aOR = 2.123; 95% CI 1.477–2.706); The level of education was associated, women with a higher level of education were significantly associated (aOR = 2.591; 95% CI 1.329–3.062).

**Table 3:** Multivariate Logistic Regression Examining Factors Associated with the uptake of Postpartum Intrauterine Contraceptive Device Use

Variable	C.O.R**. (95% CI)	***p***-value	a.O.R***. (95% CI)	***p***-Value
**Parity**				
P1	1		1	
P2-p4	3.450 (1.868–6.370)	0.001*	2.265 (1.472–3.163)	0.004*
P4	5.260 (2.472–11.194)	0.001*	3.524 (1.711–7.257)	0.001*
**Mode of delivery**				
Cesarean section	1		1	**1**
Normal delivery	0.285 (0.166–0.489)	0.001*	2.623 (2.017–6.507)	0.001*
**Pregnancy interval**				
> 24 months	1		1	1
12-24 months	1.115 (0.662–1.877)	0.682	1.517 (1.293–3.115)	0.101
6–12 months	1.934 (1.086–3.445)	0.025*	2.123 (1.477–2.706)	0.001*
**PPIUCD counselling**				
Yes	1.733 (1.919–3.265)	0.001*	2.072 (1.018–4.218)	0.001*
No	1		1	
**Age**				
35–40	4.740 (2.393–9.387)	0.001*	3.560 (1.660–7.632)	**0.001**
25–34	2.737 (1.630–4.596)	0.001*	2.515 (1.401–4.515)	**0.002**
15–24	1		1	
**Level of education**				
No formal education	1		1	
Primary	1.281 (0.776–2.116)	0.333	1.691 (0.941–3.037)	0.079
Tertiary	2.132 (1.900–3.050)	0.333	2.530 (1.401–4.260)	0.045*
**Spouse approval**				
Yes	0.388 (0.237–0.638)	0.001*	2.591 (1.485–4.492)	0.001*
No	1		1	

*p < 0.05: significant variables; **COR crude odds ratios ***aOR Adjusted odds ratio

### In-depth interviews (IDI)

In this study, midwives were providing the PPIUCD and they were supported by the Ministry of Health through training and supply for the methods at the health facility level, the investigator wanted to understand the midwives’ experiences and perceptions regarding the PPIUCD provision. A total of 10 in-depth interviews was conducted with the midwives who work on the labor suite, immediate postpartum ward, antenatal, and family planning service. From the analysis the following themes emerged:

**Table:** 

Code	Categories	Theme
ANC counselling		
Teaching mothers in labor		
Teaching mother in postpartum	Education of the mothers and their partners on PPIUCD	Counselling of couples
Teaching couple		
Discuss the method		
Tell mother about side effect		
Emphasize mother preference		
Correct myths		
Correct misconception		
Inform about availability		
**Code**	**Categories**	**Theme**
Training has shaped my ability		
Trained to provide counselling	Training health care providers on PPIUCD	Training of Midwives
Before was not familial		
Competence training		
Working with confidence		
Improved quality service		
Regular training		
**Code**	**Categories**	**Theme**
	Midwives perception and behaviors about PPIUCD provision	Midwives attitudes
Inform mothers about the method		
Hear mother preference		
Respect mothers choice		
Encourage the mother to use the method		
Tell mother about the benefits of PPIUCD		
Teach mothers on how it works		
**Code**	**Categories**	**Theme**
Doubt on the effectiveness of the method		
Resistant to use the method		
A mistaken belief that it can interfere with their life	Clients misconception on the use of PPIUCD	Clients misperception
Rumors and myths from friends and relatives		
The method will interfere with sexual intercourse		
Prefer to use other familial method use previously		
**Code**	**Categories**	**Theme**
The health facility facilitate the availability of methods		
A high number of clients with high demand	Availability and supply of commodities	Availability of contraceptives
The health facility support the supply for PPIUCD		
A regular supply for methods in labor, ANC, postpartum		
All methods are available in the health facility		
We provide postpartum FP every day		
Make sure no one miss opportunity		

### The theme I: Counselling for couples

Health care providers suggested that the health education of women and their husbands during antenatal care was important in increasing the acceptability and the use of PPIUCD. The midwives asserted that women come with wrong cultural beliefs which hinders the use of PPIUCD among postpartum women, through educating the couple during antenatal care corrected the myths and misconception regarding PPIUCD use and gave adequate time for women and their partners to decide earlier and make an informed choice on using PPIUCD before the delivery. This was evidenced in one of the quotes by health care providers below:

*“The counselling starts from antenatal care, during labor and after delivery, we teach mothers about family planning, and we emphasize a lot on family planning after delivery. Most of the women are not aware of immediate postpartum family planning, we focus on it a lot during antenatal care and give to women the time to think about it and they come to deliver when they have already made their choice, it is also the right time for us … as we have a lot of time to discuss with women, we told them that they can get the method PPIUCD within 48 hours before discharge, we explain about all side effects for the methods and let them make choice, but most of the time we emphasize a lot on methods depending on mother preference and the methods that can fit the mother”. IDI 3*

### Theme II: training for midwives

Training on PPIUCD services increases the confidence and competence among health care providers to provide the methods. The majority of participants reported that they had been trained in post-partum family planning.

*“. At the beginning of PPIUD implementation I was not comfortable and having fear of providing the method due to different side effects that can occur like perforation of the uterus, expulsion … , but now because of competence from the training we got, I provide the methods with confidence and it has improved the quality care service delivery.” IDI 3*

The effective training for a health care provider, increase the quality of provision:

*“The training effects me as a midwife I have been trained and provide PPIUD counselling, but those who have not yet trained, it’s hard for them to give, because they don’t want to provide the method they are not conversant with, and affect the acceptability and use of the method to women, the facility facilitates us in terms of the regular training provision but there is need to increase training”****.*** *(IDI 2).*

### Theme III: midwives attitudes

The midwives perceived that the use of PPIUCD was a suitable and preferred family planning method for women in the immediate postpartum period. The midwives perceived that the method was less painful for the women and that it required no additional need for frequent re-visits by the client after insertion to the hospital.

*“I teach the mother about all methods we have, I do hear her preference, and I advise her depending on her preference, I respect mother choice when I am about to provide the method. I encourage women to use the PPIUCD because it is safe and less painful insertion considering with interval PPIUD method and will not require women to comeback frequently at the hospital and this is the right time when women are still the facility, motivated with health care providers and her husband to think about her next pregnancy spacing and make informed choice”. (IDI 1.).*

### Theme IV: clients misperception

According to the midwives, women were hesitant to use the method because of the mistaken belief that it might interfere with sexual intercourse. This was captured in the following quote below:

*“When we approach women during counselling on PPIUCD, they used to doubt methods whether it will work, different rumors and myth from their friends and relatives about PPIUD, have confusion whether her husband will be comfortable during sexual intercourse and because they are not familiar with using method immediately after delivery as they were routinely used to injectable and pills after six weeks or before based on this myth” (IDI 5).*

### Theme V: availability of contraceptives

The availability and supply of commodities increase the consumption of a service. The midwives recognized that regular supply of the PPIUCD to ensure that no woman would miss getting the method because of stock out.

*“The hospital administration ensures that all the training materials and the PPIUCD methods are available in the hospital. We have a high number of deliveries here at Muhima hospital, and there is a high demand for the method before discharge as women got counselling from ANC. The administration supports us in all ways to help us deliver the PPIUCD methods to women. They provide regular supply for the methods in labor and family planning services to make sure no woman can miss the opportunity for PPIUD insertion” (IDI 7).*

## Discussion

The findings of the study showed that the uptake of PPIUCD was associated with normal birth, PPIUCD counselling, spousal approval, parity, birth interval, level of education. Health education of mothers and partners on PPIUCD, training of health care providers, and availability of supplies to provide PPIUCD influenced use PPIUCD.

Overall the prevalence rate of 28.1% for the use PPIUCD in this study was generally higher than other study done in Ethiopia 12.4%, in India 12.6%, Texas in 13.5% Brunson et al.,; Gonie et al.,; Rajasthan et al.,, [11, 17] and this prevalence was in line with findings from Tanzania which found 27% [18] and was attributed on the fact that the health care providers from Tanzania setting were trained to provide PPIUCD and enhance women knowledge during ANC. The high prevalence rate in Rwanda could be attributed to the fact that Muhima Hospital is one of the piloting health facility for PPIUCD implementation in Rwanda and the Ministry of Health has put many resources such training of health care providers, supplying the methods in the facility which might have increased the use of the PPIUCD. The higher use of PPIUCD in the facility could be due attributed to the regular provision of all the resources required for the training of midwives as well as regular supplies of IUD materials in the health facility. This shows that if there is a commitment from the government, there is the sustainability of any outcome.

The level of education influences health behavioral, in this study, it was observed that the use of immediate PPIUCD increased with the level of education. Women who are educated are empowered to decide fertility control and can better understand the health information offered to them regarding the use of PPIUCD It was also reported by midwives that women who are educated were more likely to visit a health facility and receive counselling for different methods available in the health facility. This is in line also with a study done in Ethiopia where women with formal education were more likely to use PPIUCD compared with counterparts [9].

In this study Counselling for mothers during prenatal visits increased the awareness for mothers regarding the PPIUCD, Counselling the couple during antenatal was significantly associated with the use of PPIUCD, which was consistent with the various studies done in West-central Africa and Ethiopia [16, 19]. The role of counselling was supported by midwives who reported that women couselled during antenatal care increased the demand and uptake of PPIUD because this creates an opportunity to have the mothers and their partners together.. Furthermore, the antenatal counselling also allows the client to have enough time to discuss with the family methods and also to have access information from health care providers which enables them to make an informed decision before the time of delivery as women may have more information on the benefits of initiating postpartum contraceptives utilizations on a timely manner and this can increase their intention to use immediately after delivery.

Male involvement in reproductive health is an essential component in promoting maternal and family health. In this study, women who received approval to use the method from their partners were more likely to use the method than their counterparts. Additionally, the midwives mentioned that when husbands/ partners attend the ANC, they understand well the role of initiating contraceptives immediately after birth and make it easy to decide at home before coming to the facility. This was similar to the study done in Ethiopia which showed that the higher odds of women who accept the PPIUCD were women who have had a discussion with their partners [20] and a study in India [21] reported that 42.96% of women declined to use PPIUCD due to partners’ non-involvement in decision making. This indicates that the male’s involvement in the decisions making process is necessary to increase the use of PPIUCD and prolong the continuation of the method and also it should be explained the importance of male involvement during counselling and decisions making concerning to fertility and reproductive health decisions and the need for involving partners in issues related to health and specifically reproductive health.

To achieve optimal birth spacing and ultimately to improve birth outcomes, there is a need to have access to postpartum family planning services after birth. The short birth to pregnancy interval less than two years was significantly associated with the use of PPIUCD. These findings were in line with a study done in India [22] who got the same findings such as among women with short birth interval were more likely to use PPIUCD, this was also supported by World Health Organisation guidelines released that healthy timing and spacing of pregnancies have a positive effect on maternal health and newborn outcomes, when promoted in countries with high birth rates, 32% of all maternal deaths and over 1 million deaths of children under five could be prevented [23].

The number of children has been identified as an important individual characteristic influencing women’s reproductive health behaviors including uptake for postpartum family planning. The parity was associated with the use PPIUCD, women with a higher parity were more likely to use PPIUCD, this simply reflects that women with high parity required long term contraceptives for spacing secondly women who are multipara were more likely to meet the health service provider and obtained suitable information regarding mode of family planning. The findings were consistent with the study done in Ghana which reported a high acceptance rate to use PPIUCD among multipara women [24].

The mode of delivery either spontaneous vaginal delivery or cesarean section contributes to PPIUCD insertion, in this study women who delivered normally were more likely to use PPIUCD compared to women who had a caesarian section. The higher utilization of PPIUCD among women with normal delivery could partly be attributed to the fact that during the training sessions a lot of emphases were put on the midwives and the obstetricians were not trained so they lacked the knowledge and information. There is a need to train both Midwives and obstetricians so that the service could be provided by both. In India, both midwives and obstetricians were trained and the findings from the study reported that 83% of PPIUD were inserted during normal delivery [25].

The findings from health care providers interview show that various measures need to be considered for effective PPIUCD delivery to the mothers who are in need including; the training of health services, the availability of the method in facility, providers respects to women, privacy, waiting and counseling time were predictors for PPIUCD use, this shows that the facility needs to make regular training and supply of the method at the facility level, these predict the use of the PPUCD and increases the provider confidence to counsel and to provide the method and hence it impacts the client satisfaction. Similar findings were reported by Melissa et al., which shows that the competence of the health service provider is linked to the client’s satisfaction [26].

This study has explored experience and perceptions for services providers offering PPIUCD services at Muhima hospital, the study has highlighted the perception and experiences of providers about PPIUCD. The participants show positive perceptions and experiences about PPIUCD which can be a critical advocate for these methods in all levels of facilities across the country.

The current study shows that participants were comfortable with counselling the clients about PPIUCD; during antenatal visits, labor, and immediately after delivery. This is due to that majority of providers from the facility participating in this study have got the competence training which helped them to correct the myth and misconception related to PPIUCD to help women understand the method, this finding is in line with guidelines from (K4health 2014), recommending that health care providers competence is critical ineffective service delivery for effective use [27]. The ANC period was a suitable period for counseling the women on PPIUCD. The reason stated by midwives was that it gives time for reinforcement of the messages and allows enough time for the client to discuss with family to arrive at an informed decision after delivery, this finding is in line with valliappan et al. (2018) study done in Indi [12], stated that health care providers should continue health counseling to the pregnant women, especially in the last trimester and in every visit to reinforce the uptake for PPIUCD [12].

Additionally, other participants show that the immediate PPIUCD counselling after delivery was also suitable for counselling as the women are most receptive to family planning during this period, the implication of counselling during this period define that mother is more likely to accept a method as she is motivated and does not want to get pregnant soon, provide a reliable contraceptive method and ensures that women leaves the facility safe and protected from pregnancy in the near the future, this finding is in line with the study done by Melissa et al. 2012 to assess uptake for PPFP and shows that women choose PPIUCD due is the long-term method and safe [28].

The findings from this study show that the training of health services and the availability of the method in facility providers respect to women, privacy, waiting, and counselling time were predictors for PPIUCD use, this shows that the facility needs to make regular training and supply of the method at the facility level, these predict the use of the PPUCD and increases the provider confidence to counsel and to provide the method thus impact the client satisfaction; similar findings were reported by Melissa et al. (2012) show that the competence of health service provider is linked to client’s satisfaction [28].

### Study strength and limitation

- This study used a mixed-method approach that provided complementary findings. The weaknesses inherent in the quantitative or qualitative approach were supplemented by findings from either of the approaches.
- The study has used a facility-based cross-sectional design, the investigator was able to collect data from the client at one point in time regarding the uptake for the method, however, there were no follow up made to assess the side effects of the method and client satisfaction.

## Conclusion

Over a quarter (28%) of women in the postpartum period used PPIUCD. The use of PPIUCD was associated with spousal approval, normal vaginal birth, antenatal counselling for PPIUCD, higher parity, short birth interval, and level of education. Health education of women and partners on PPIUCD, training of health providers, misconceptions regarding PPIUCD use and availability of supplies to provide PPIUCD influenced the use of PPIUCD.

## Data Availability

The datasets used and /or analyzed during the current study are not publicly available due to some privacy reasons but are available from the corresponding author on reasonable request.

## Abbreviations

ACOG: American College of Obstetricians and Gynecologists
ANC: Antenatal Care
DHS: Demographic and Health Survey
FGDs: Focus Group Discussions’
FIGO: International Federation for Gynecologist and Obstetricians
IUDs: Intrauterine Devices
LARC: Long-acting reversible contraception
MOH: Ministry Of Health
NISR: National Institute of Statistics of Rwanda
PPFP: Postpartum Family planning
PPIUCD: Post-Partum Intrauterine Contraceptive Devices
TWFR: (Total Wanted Fertility Rate)
UNFPA: United Nations Fund for Populations Activities
USAID: United State Agency for International Development
WHO: World Health Organization
